# Cultured cells of the blood–brain barrier from apolipoprotein B-100 transgenic mice: effects of oxidized low-density lipoprotein treatment

**DOI:** 10.1186/s12987-015-0013-y

**Published:** 2015-07-17

**Authors:** Nikolett Lénárt, Fruzsina R Walter, Alexandra Bocsik, Petra Sántha, Melinda E Tóth, András Harazin, Andrea E Tóth, Csaba Vizler, Zsolt Török, Ana-Maria Pilbat, László Vígh, László G Puskás, Miklós Sántha, Mária A Deli

**Affiliations:** Laboratory of Animal Genetics and Molecular Neurobiology, Institute of Biochemistry, Biological Research Centre, Hungarian Academy of Sciences, Temesvári krt. 62, 6720 Szeged, Hungary; Biological Barriers Research Group, Institute of Biophysics, Biological Research Centre, Hungarian Academy of Sciences, Szeged, Hungary; Laboratory of Molecular Stress Biology, Institute of Biochemistry, Biological Research Centre, Hungarian Academy of Sciences, Szeged, Hungary; Laboratory of Functional Genomics, Laboratories of Core Facilities, Biological Research Centre, Hungarian Academy of Sciences, Szeged, Hungary; Laboratory of Neuroimmunology, Institute of Experimental Medicine, Hungarian Academy of Sciences, Budapest, Hungary

**Keywords:** Apolipoprotein B-100, Transgenic mouse, Blood–brain barrier, Brain endothelial cell, Pericyte, Glial cell, Oxidized low density lipoprotein, Reactive oxygen species, Permeability, Membrane fluidity

## Abstract

**Background:**

The apolipoprotein B-100 (ApoB-100) transgenic mouse line is a model of human atherosclerosis. Latest findings suggest the importance of ApoB-100 in the development of neurodegenerative diseases and microvascular/perivascular localization of ApoB-100 protein was demonstrated in the cerebral cortex of ApoB-100 transgenic mice. The aim of the study was to characterize cultured brain endothelial cells, pericytes and glial cells from wild-type and ApoB-100 transgenic mice and to study the effect of oxidized low-density lipoprotein (oxLDL) on these cells.

**Methods:**

Morphology of cells isolated from brains of wild type and ApoB-100 transgenic mice was characterized by immunohistochemistry and the intensity of immunolabeling was quantified by image analysis. Toxicity of oxLDL treatment was monitored by real-time impedance measurement and lactate dehydrogenase release. Reactive oxygen species and nitric oxide production, barrier permeability in triple co-culture blood–brain barrier model and membrane fluidity were also determined after low-density lipoprotein (LDL) or oxLDL treatment.

**Results:**

The presence of ApoB-100 was confirmed in brain endothelial cells, while no morphological change was observed between wild type and transgenic cells. Oxidized but not native LDL exerted dose-dependent toxicity in all three cell types, induced barrier dysfunction and increased reactive oxygen species (ROS) production in both genotypes. A partial protection from oxLDL toxicity was seen in brain endothelial and glial cells from ApoB-100 transgenic mice. Increased membrane rigidity was measured in brain endothelial cells from ApoB-100 transgenic mice and in LDL or oxLDL treated wild type cells.

**Conclusion:**

The morphological and functional properties of cultured brain endothelial cells, pericytes and glial cells from ApoB-100 transgenic mice were characterized and compared to wild type cells for the first time. The membrane fluidity changes in ApoB-100 transgenic cells related to brain microvasculature indicate alterations in lipid composition which may be linked to the partial protection against oxLDL toxicity.

**Electronic supplementary material:**

The online version of this article (doi:10.1186/s12987-015-0013-y) contains supplementary material, which is available to authorized users.

## Background

Cardiovascular diseases are the leading cause of death accounting for 48% of deaths worldwide. The main risk factor for cardiovascular diseases is atherosclerosis, which is associated with a permanently high level of serum very low density and low density lipoproteins (VLDL and LDL). Apolipoprotein B-100 (ApoB-100) is a 512 kDa glycoprotein, and the main apoprotein component of VLDL and LDL particles which play a key role in the plasma lipid transport. Although earlier apolipoprotein and cholesterol research was mainly focused on cardiovascular diseases, latest findings indicate that apoB-100 might be also involved in the development of neurodegenerative disorders. Recent studies show that the human neurodegenerative disorder, Alzheimer’s disease, is accompanied by elevated ApoB concentration in the serum [1, 2] and a high serum level of ApoB-100 modulates cerebral amyloid deposition in vivo [3].

Previously, we have generated a transgenic mouse line overexpressing the human ApoB-100 protein [4]. This strain showed a significantly elevated serum triglyceride level on normal chow diet, and a significantly increased serum cholesterol level when fed on high fat diet compared to wild-type littermates [5]. The hypercholesterolemic ApoB-100 transgenic strain is frequently used as a model of atherosclerosis [6, 7]. Recently, we have shown that chronic hypertriglyceridemia led to the development of neurodegenerative changes in brains of ApoB-100 transgenic mice. These changes include hyperphosphorylation of tau protein at several sites, extensive cortical and hippocampal neuronal apoptosis, marked reduction in the number and size of the dendritic spines in the hippocampal neurons and impaired hippocampal presynaptic functions [8]. Moreover, significant enlargement of brain ventricles in a transgene dose dependent manner was demonstrated by magnetic resonance imaging [9]. We have also shown that there is an extensive lipid peroxidation in the cortex and hippocampus of transgenic animals [10]. Besides the above mentioned neuropathological changes, a large decrease in the density of cortical microcapillary network was observed in transgenic compared to wild-type mice [11].

In spite of the well-known contribution of ApoB-100 to the development of hyperlipidemia and atherogenesis, there is less information on its presence and potential function in the brain. Previously, we have demonstrated that ApoB-100 protein is present in the cerebral cortex of mice overexpressing ApoB-100 [8] and shows microvascular/perivascular localization [10]. However, little is known about the effect of ApoB-100 on the cells of the blood–brain barrier (BBB).

The aim of this study was to provide information on the expression and presence of ApoB-100 in endothelial cells, glial cells and pericytes in the cerebrovascular system in wild-type and transgenic mice. The effect of ApoB-100 on oxidized low-density lipoprotein (oxLDL)-induced cytotoxicity and on membrane fluidity of cerebral endothelial cells using various in vitro experimental approaches was investigated.

## Methods

### Materials

All reagents were purchased from Sigma-Aldrich Ltd., Hungary, unless otherwise indicated.

### Animals

All animal studies were conducted following the 1998. XXVIII. Hungarian law and the EU Directive 2010/63/EU about animal protection and welfare. For animal experiments official approvals have been obtained from our local animal health authority, the Governmental Office for Csongrád County, Directorate of Food Chain Safety and Animal Health (Permit numbers: XVI.02752/2009 and XVI./834/2012).

For in vitro studies primary cells were obtained from the ApoB-100 transgenic mouse line, a mouse model of human atherosclerosis [4]. F1 transgenic offsprings were backcrossed six times with C57BL/6J mice to ensure a more homogenous genetic background for appropriate comparative studies. Non-transgenic littermates were used as wild-type controls. Previously, it was demonstrated that these transgenic animals fed on high cholesterol diet developed severe coronary atherosclerosis by the age of 6 months [9]. Therefore during our experiments all mice were at least 6 months old. Animals were fed on regular standard rodent chow and water ad libitum and kept under a 12 h light/12 h dark cycle. During the experiments all efforts were made to minimize animal suffering and pain.

### In vitro mouse blood–brain barrier model

Primary mouse brain endothelial cell isolation was performed using 6-month-old C57BL/6 wild type and transgenic mice based on methods from our group [12–14]. Animals were sacrificed with cervical dislocation. Mouse forebrains were collected in ice-cold sterile phosphate buffered saline (PBS). Meninges were removed using sterile filter paper and the tissue was cut into 1 mm^3^ using a scalpel and digested with enzymes (1 mg/ml collagenase type II and 15 µg/ml deoxyribonuclease type I, Roche, Switzerland) in Dulbecco’s modified Eagle medium (DMEM/F12, Gibco, Life Technologies, USA) at 37°C for 50 min. Microvessels were separated from myelin by centrifugation on a 20% bovine serum albumin (BSA)-DMEM gradient (1,000×*g*, 20 min, 3 times). The collected vessels were further digested by enzymes (1 mg/ml collagenase–dispase and deoxyribonuclease type I in DMEM, both from Roche) for 35 min. Digested cerebral vessels were washed three times in cell culture medium then seeded onto Petri dishes (60 mm; Orange Scientific, Belgium) coated with collagen type IV and fibronectin. Mouse brain endothelial cells were cultured in DMEM/F12 supplemented with plasma-derived bovine serum (15%; First Link, UK), heparin (100 µg/ml), basic fibroblast growth factor (1 ng/ml; Roche), insulin (5 µg/ml), transferrin (5 µg/ml), sodium selenite (5 ng/ml), and gentamycin (50 µg/ml). Cells were grown under selective culture conditions for the first 4 days. Culture medium contained puromycin (3 µg/ml) to eliminate P-glycoprotein negative contaminating cell types [15]. When endothelial cells reached 90% confluency on the 4th–5th day after seeding to the Petri dish, cells were subcultivated into 96-well plates (Corning, USA; ACEA, USA), cover slips (VWR, USA) and hanging cell culture inserts for different experiments, respectively. All surfaces were coated with collagen type IV and fibronectin. Endothelial cells were passaged at a cell number of 5 × 10^3^ cells/well for 96-well plates and 3.5 × 10^4^ cells/cover slips. Since primary brain endothelial cell isolation yield from mice is low compared to other species, 24-well format culture inserts were used for permeability studies (ThinCert, 24-well format, polyethylene terephthalate membrane, 0.33 cm^2^ surface, 3 µm pore size, Greiner Bio-one, Germany). These culture inserts were found to be appropriate for BBB permeability studies [16]. For barrier integrity tests endothelial cells were seeded to the luminal side of culture inserts at a cell number of 2.5 × 10^4^ cells/insert. To enhance BBB characteristics, primary mouse endothelial cells were co-cultured with primary mouse cerebral glial cells and pericytes [17].

Primary cultures of mouse pericytes were prepared by the same protocol as primary mouse brain endothelial cells, except for a shorter time of the second enzymatic digestion (15 min) and the omission of the puromycin treatment. At the end of the isolation process cerebral microvessels containing pericytes beside endothelial cells were seeded into collagen type IV coated 60 mm culture dishes (Orange Scientific, Belgium). After 4 days of culture in DMEM, 10% fetal bovine serum (FBS), and gentamycin (50 µg/ml) attached cells reached 70% of confluency. Pericytes were passaged into bigger, uncoated dishes (Orange Scientific, Belgium). Mouse pericyte cultures were used at second passage and seeded at a cell number of 5 × 10^3^ cells/well for 96-well plates, 3 × 10^4^ cells/cover slips and 5 × 10^3^ cells/culture inserts. Primary mouse pericytes stain positive for α-smooth muscle actin but not for von Willebrand factor or glial fibrillary acidic protein (GFAP) [17, 18].

Primary mouse glial cells were obtained from 1 or 2-day-old wild type or ApoB-100 transgenic mice [12, 13]. Meninges were removed by fine forceps from brains. Little pieces of cortices were minced and mechanically dissociated by pressing the tissue through a nylon mesh (40 µm, Millipore, USA). Cell clusters were seeded onto uncoated 75 cm^2^ flasks (TPP, Germany) and cultured in DMEM containing FBS (10%; Lonza, Switzerland) and gentamycin (50 µg/ml) until 90% confluency. Glial cells were passaged at a cell number of 5 × 10^3^ cells/well for 96-well plates, 3 × 10^4^ cells/cover slips and 10^5^ cells/wells for 24-well plates (Greiner Bio-One, Germany). For the BBB co-culture model mixed glial cells were cultured for 2 weeks before use. Confluent glia cultures contained 90% of astroglia (positive for GFAP), and 10% microglia (positive for CD11b).

To establish the triple co-culture BBB model culture inserts were put into 24-well plates containing confluent glia cultures. Mouse pericytes were subcultured to the under side of the culture membranes and mouse brain endothelial cells were passaged to the upper side of the coated inserts. Both compartments received endothelial culture medium [17]. After 2 days of co-culture, cells were kept in culture medium containing 550 nM hydrocortisone and 10 mM lithium chloride [19, 20].

### Treatment

Mouse endothelial cells, pericytes and glial cells were treated with native LDL (200 µg/ml) or oxLDL (50–200 µg/ml) for 1 and 24 h in serum-free culture medium containing 1% BSA (fatty acid free) to avoid artefacts or biases caused by the lipid content of the serum. Both native and oxLDL were purified from human plasma and purchased from Biomedical Technologies Inc. (USA). In viability assays, Triton X-100 detergent was used at 10 mg/ml concentration as a reference compound to cause cell death. For reactive oxygen species assay hydrogen peroxide (H_2_O_2_) and for the nitric oxide assay sodium nitroprusside (SNP) were used as positive controls. In all experiments, treatments were carried out carefully not to disturb cell monolayers when changing the medium.

### Cell viability assays: real-time monitoring of impedance and lactate dehydrogenase release

Impedance-based cell electronic sensing is a label-free, non-invasive technique for dynamic monitoring of the biological status of living cells. The RTCA-SP instrument (ACEA Biosciences, Inc., USA) registers the impedance of adherent cells automatically and continuously to quantify cell proliferation and viability in real-time [21, 22]. E-plates (96-well culture plates with built in gold electrodes) were coated with collagen type IV and fibronectin for brain endothelial cells or with rat tail collagen for pericytes and glial cells at room temperature and dried for 40 min under UV and air-flow. Culture medium (60 μl) was added to each well for background measurements, then 40 µl of cell suspension was pipetted into the wells. Cell density was 5 × 10^3^ cells/well for all cell types. Cell growth was monitored until cells reached a steady phase, when they were treated with LDL or oxLDL. Impedance was monitored every 10 min. The cell index at each time point was defined as (Rn − Rb)/15, where Rn is the cell-electrode impedance of the well when it contains cells and Rb is the background impedance of the well with the medium alone.

The presence of the intracellular enzyme, lactate dehydrogenase (LDH) in the culture supernatant is an indicator of cell membrane damage. The concentration of LDH was determined using a commercially available kit (Cytotoxicity detection kit LDH, Roche, Switzerland). For LDH release assay brain endothelial cells, pericytes or glial cells were cultured in 96-well plates. After treatments culture supernatants (40 µl) were collected into another 96-well plate and were incubated with equal amounts of reaction mixture for 20 min on a horizontal shaker. The enzyme reaction was stopped by 0.1 M HCl. Absorbance was measured at a wavelength of 492 nm with a multiwell plate reader (Fluostar Optima, BMG Labtechnologies, Germany). Cytotoxicity was calculated as percentage of the total LDH release from cells treated by 1% Triton X-100 detergent.

### Detection of reactive oxygen species and nitric oxide production

For the measurement of reactive oxygen species (ROS) and nitric oxide (NO) production fluorometric detection probes were used. ROS was detected by chloromethyl-dichloro-dihydro-fluorescein diacetate (DCFDA) and NO was detected by 4-amino-5-methylamino-2′,7′-difluorofluorescein diacetate (DAF-FM, both from Molecular Probes, Life Technologies, USA). These indicators penetrate the cell membrane by diffusion and interact with intracellular esterases to be activated. After DCFDA oxidation or reaction of DAF-FM with NO, fluorescent molecules are produced, and their signal is detectable and proportional to the produced ROS or NO. Confluent brain endothelial cells, pericytes or glial cells and were cultured in 96-well black-walled plates. After treatment cells were incubated with Ringer-Hepes buffer (118 mM NaCl, 4.8 mM KCl, 2.5 mM CaCl_2_, 1.2 mM MgSO_4_, 5.5 mM d-glucose, 10 mM Hepes, pH 7.4) containing 2 µM DCFDA or 2 µM DAF-FM and 1.5 µM pluronic acid (Life Technologies, Molecular Probes, USA) for 1 h at 37°C. Hydrogen peroxide treatment (100 µM) served as a positive control in the ROS assay and sodium nitroprusside (60 µM) treatment for the NO production. Fluorescence was measured by Fluostar Optima multiwell plate reader (BMG Labtechnologies, Germany) at 485 nm excitation and 520 nm emission wavelengths every 5 min for 1 h. Fluorescent values are presented as a percentage of the control group (cells receiving only treatment medium).

### Measurement of barrier functions: resistance and permeability

Transendothelial electrical resistance (TEER) is an indicator of the permeability of interendothelial tight junctions; therefore it is a key parameter to validate BBB tightness in cell culture models. High TEER indicates models with a good paracellular barrier which can be used for permeability and transport experiments. TEER was measured by an EVOM resistance meter with chamber electrodes appropriate for 24-well culture inserts (World Precision Instruments, USA) and it was expressed relative to the surface area of the monolayers (Ω cm^2^). TEER was measured every day and the resistance value of cell-free inserts was subtracted from the measured values. Cells were treated with LDL and oxLDL on the 4th day of co-culture. The transendothelial penetration of Evans blue-labeled albumin (M_W_ 67 kDa) was measured across the monolayers as previously described [12, 13]. Evans blue labels albumin effectively and can be used for tracking the penetration of albumin in tissues and across cell layers [23]. For permeability experiments, culture inserts were placed to 24-well plates (Greiner, Austria) containing 530 µl Ringer-Hepes buffer. In the upper compartment culture medium was replaced by 70 µl Ringer-Hepes solution containing 1% BSA labeled with 165 µg/ml Evans blue. The plates with inserts were slowly agitated using a horizontal shaker (100 rpm; Biosan, Latvia) in the CO_2_ incubator for 1 h. During the assay at 20, 40 and 60 min, inserts were moved to a new well containing Ringer Hepes buffer. Samples from the upper (luminal) and lower (abluminal) compartments were collected and the marker molecule content was measured at 584 nm excitation and 680 nm emission wavelengths (Fluostar Optima, BMG Labtechnologies, Germany). The albumin flux was also measured across cell-free inserts. Endothelial permeability coefficient (Pe) was calculated as previously described [12, 24]. Clearance was expressed as μl of luminal compartment volume from which the tracer is completely cleared. The cleared volume was calculated from the concentration (C) of the tracer in the luminal and abluminal compartments and the volume (V) of the abluminal compartment (530 µl) by the following equation:

$${\text{Cleared}}\,\, {\text{volume} }(\upmu {\rm l}) = \frac{C_{abluminal} \times V_{abluminal}}{C_{luminal}}$$

The average cleared volume was plotted vs. time, and permeability-surface area product (PS) values for endothelial monolayers (PSe) were evaluated by the following equation:

$$\frac{1}{{PS_{endothelial} }}\;\, = \frac{1}{{PS_{total} }}\quad - \quad \frac{1}{{PS_{insert} }}\;$$

PSe divided by the surface area (0.33 cm^2^) generated the Pe (10^−6^ cm/s).

### Immunohistochemistry and quantification

Morphological comparison of primary cells derived from wild type and ApoB transgenic mice was performed using immunohistochemical staining. Endothelial cells were labeled for claudin-5, occludin, zonula occludens protein 1 (ZO-1) and β-catenin, pericytes for α-smooth muscle actin (α-SM) and astroglia for glial fibrillary acidic protein (GFAP). All cell types were also characterized for the presence of ApoB. Cells were grown on rat tail collagen coated glass coverslips (1 cm^2^, borosilicate, VWR, USA). For claudin-5, ZO-1 and β-catenin staining, cells were fixed with cold acetone-methanol (1:1) for 10 min. For occludin, GFAP, α-SM and ApoB labeling cells were fixed with 3% paraformaldehyde for 30 min at room temperature and permeabilized with 0.5% Triton-X100. After fixation and permeabilization, cells were washed three times with PBS and non-specific binding sites were blocked with 3% BSA-PBS for 1 h at room temperature for all antibodies except for ApoB, for which cells were blocked with 5% rabbit serum (Dako, Denmark). Incubation with rabbit-anti-claudin-5, rabbit-anti-ZO-1, rabbit-anti-occludin (Life Technologies, Invitrogen, USA), mouse-anti-α-SM (Dako, Denmark), goat-anti-hApoB (Millipore, Germany), rabbit-anti-β-catenin and mouse-anti-GFAP primary antibodies lasted overnight at 4°C. Cells were incubated with anti-rabbit secondary antibodies labeled with CY3 or Alexa Fluor 488 (Life Technologies, USA), anti-mouse secondary antibodies Alexa Fluor 555 or 488 (Life Technologies), anti-goat Alexa Fluor 488 (Jackson ImmunoResearch Labs, USA) and H33343 dye to stain nuclei for 1 h at room temperature. Between incubations cells were washed three times with PBS. Coverslips were mounted in Fluoromount-G (Southern Biotech, USA). Stainings were visualized by a Leica TCS SP5 confocal laser scanning microscope (Leica Microsystems, Germany). At least eight non-overlapping pictures were captured for each cell type and staining from both wild type and transgenic mice. Digital image analysis (512 × 512 pixels, n = 8–16/group) was performed by using ImageJ, public domain software developed by the National Institutes of Health (USA) [13]. Immunostained areas were defined by the threshold feature of ImageJ. After subtraction of the background fluorescence (subtraction of gray values of non-expressing pixels from every pixel) in the respective channel mean grayscale value was calculated and averaged.

Endothelial cells treated with 0, 100 and 200 µg/ml oxLDL for 24 h were also stained for junctional proteins claudin-5, occludin and β-catenin as described above. Double fluorescent nucleus staining by bis-benzimide and ethidium-homodimer-1 was performed on living cells as published in [22].

### Measurement of plasma membrane fluidity in primary mouse brain endothelial cells

Brain endothelial cells from wild type and ApoB-100 transgenic mice were treated overnight with 10 µg/ml LDL or 10 µg/ml oxLDL. Control cells received culture medium. After treatment, cells were collected by trypsinization, washed once with PBS, resuspended in Ringer-Hepes buffer and counted. The density of the cells for the membrane fluidity tests was optimized by absorbance measurement to OD_360_ = 0.05 (Hewlett Packard 8452A Diode Array Spectrophotometer). Cells were labeled with 0.2 μM TMA-DPH (1-(4 trimethylammoniumphenyl)-6-phenyl-1,3,5-hexatriene; Life Technologies, USA). Fluorescence anisotropy was measured on a T-format fluorescence spectrometer (Quanta Master QM-1, Photon Technology International, USA). Excitation and emission wavelengths were 360 and 430 nm (6 nm slits). Cells were kept under a continuous stirring at 37°C [25, 26]. Anisotropy data were acquired in every second for 10 min. After measuring baseline anisotropy, we introduced a strong membrane fluidizer, benzyl alcohol (30 mM, Merck, Germany) as a positive control. Transgenic and wild type data were calculated and plotted as treatment vs. fluorescent anisotropy.

### Statistical analysis

Data are presented as mean ± SD. Statistical significance between treatment groups was determined using one-way or two-way ANOVA following Dunnett or Bonferroni post-tests (GraphPad Prism 5.01; GraphPad Software, USA). Changes were considered statistically significant at *p* < 0.05 (*); *p* < 0.01 (**) and *p* < 0.001 (***). All experiments were repeated at least twice and the number of parallel samples varied between 4 and 8.

## Results

### Characterization of cultured brain endothelial cells, pericytes and glial cells from wild type and ApoB-100 transgenic mice by immunohistochemistry

**Figure 1:**
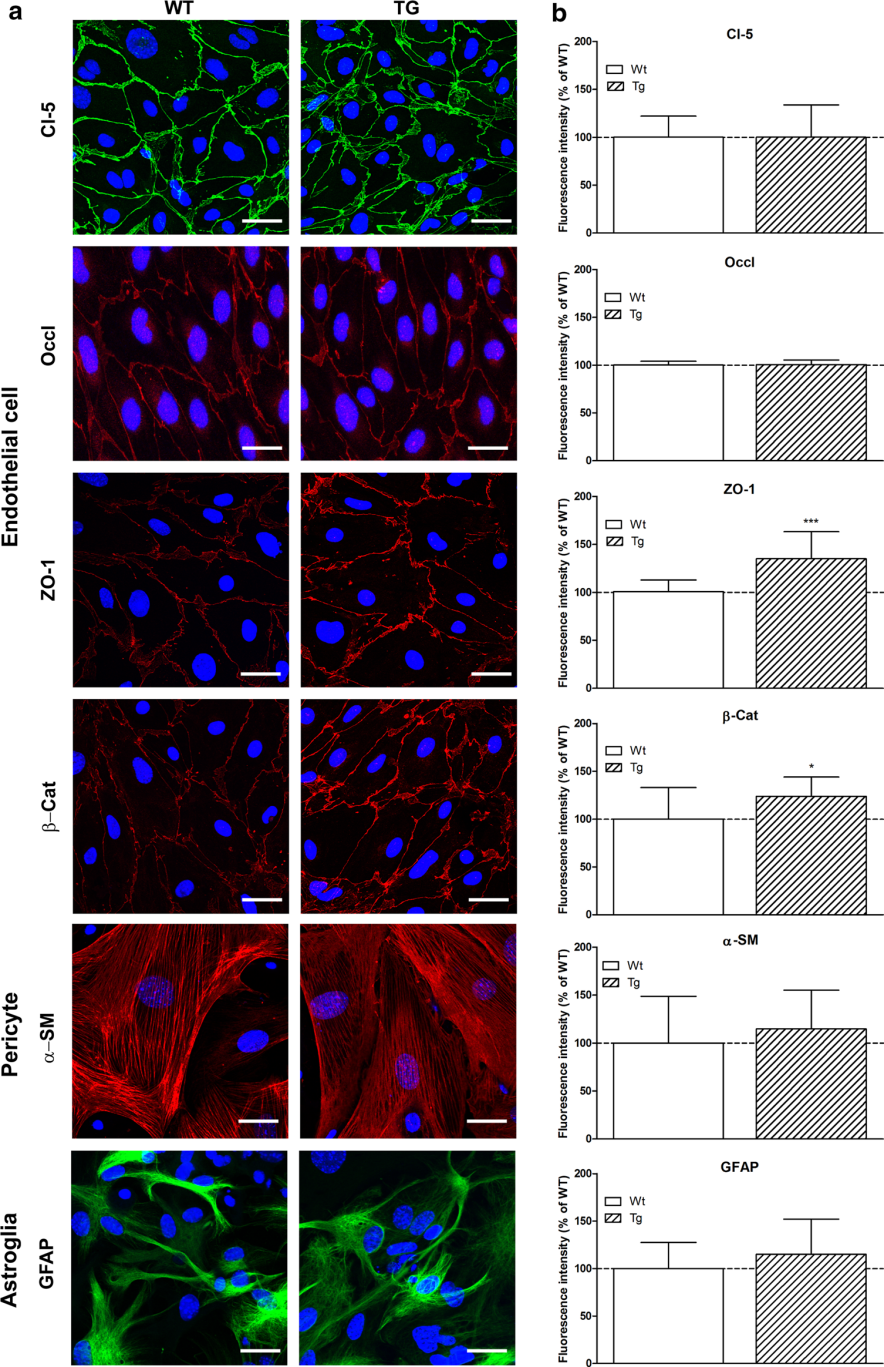
Characterization of cultured brain microvascular cells from wild type (Wt) and ApoB-100 transgenic (Tg) mice. **a** Immunostaining of brain endothelial cells for transmembrane junctional protein claudin-5 (Cl-5) and occludin (Occl), and cytoplasmic linker proteins β-catenin (β-cat) and zonula occludens-1 (ZO-1). Astroglia cells were labeled for glial fibrillary acidic protein (GFAP), pericytes were stained for α-smooth muscle (α-SM) actin. *Blue* cell nuclei. *Scale bar* 15 µm. **b** Fluorescent intensity evaluation of immunostainings with ImageJ software shown as percentage of the labeling intensity of wild type cells. Values presented are mean ± SD, n = 8–16. Statistical analysis: two-way ANOVA followed by Bonferroni post-test, *p < 0.05, ***p < 0.001 wild type compared to transgenic group.

Brain endothelial cells showed typical microvascular elongated morphology and formed confluent layers. Strong immunostaining was obtained for transmembrane tight junctional proteins claudin-5 and occludin, and cytoplasmic linker proteins ZO-1 and β-catenin in both groups (Figure 1). Junctional labeling localized to endothelial cell borders and cell–cell attachment was continuous. Pericytes were characterized by their large cell size, polygonal cell shape, branched morphology and positive staining for α-SM actin. Actin bundles were clearly visible and typical for cultured brain pericytes [17, 27]. Astroglia cells expressing GFAP showed a multipolar shape and long processes. This stellate shape was present in both wild type and transgenic groups. Cellular morphology and immunostaining intensity of pericytes and glial cells from wild type animals were similar to those from ApoB-100 transgenic mice. There were no significant differences in claudin-5 immunolabeling comparing wild type cells with ApoB-100 transgenic brain endothelial cells. The fluorescence intensity of cytoplasmic linker proteins ZO-1 and β-catenin staining was significantly higher in the transgenic group than in wild type endothelial cells (Figure 1).

**Figure 2:**
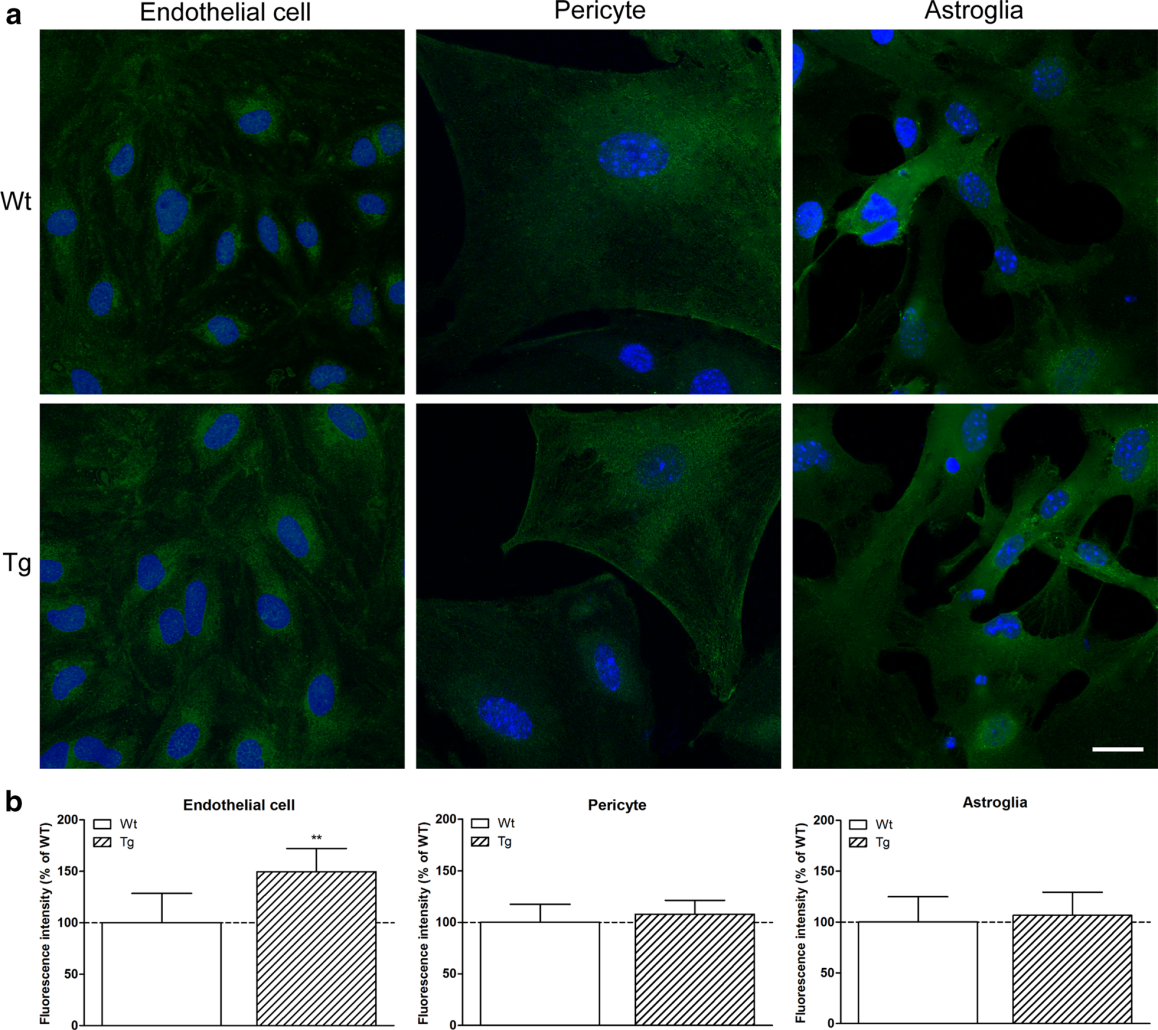
ApoB-100 immunohistochemistry in cultured brain microvascular cells from wild type (Wt) and transgenic (Tg) mice. **a** Immunostaining for ApoB-100 in brain endothelial cells, pericytes and astroglia cells. *Green* ApoB-100 staining. *Blue* cell nuclei. *Scale bar* 15 µm. **b** Fluorescent intensity evaluation for ApoB-100 immunostaining with ImageJ software shown as percentage of the labeling intensity of wild type cells. Values presented are mean ± SD, n = 10–15. Statistical analysis: two-way ANOVA followed by Bonferroni post-test, **p < 0.01 wild type compared to transgenic group.

All three cell types related to brain microvasculature were stained for ApoB, suggesting the presence of the protein (Figure 2). Quantification revealed a significantly stronger staining intensity in brain endothelial cells from transgenic mice as compared to wild type. No such difference was detected in the other two cell types.

### Effects of oxidized LDL on viability of cultured brain endothelial cells, pericytes and glial cells from wild type and ApoB-100 transgenic mice

**Figure 3:**
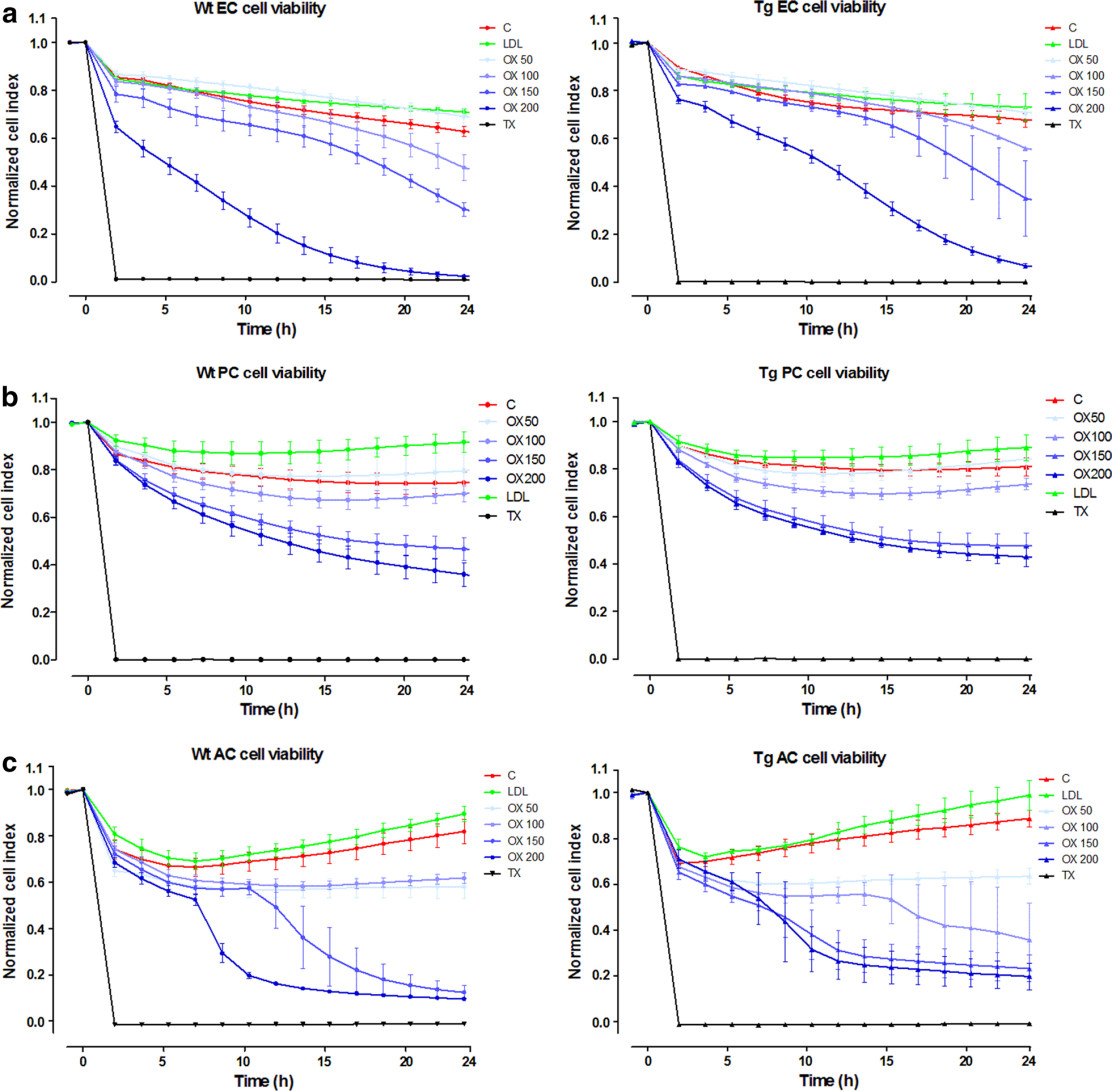
Effects of low density lipoprotein (LDL) and oxidized LDL on cell viability: impedance measurement. Effects of LDL or oxLDL treatment on the cell viability of wild type (Wt) and transgenic (Tg). **a** Primary mouse brain endothelial cells (EC), **b** pericytes (PC) and **c** astroglia cells (AC) over 24 h. Impedance is calculated from the normalized cell index which reflects cell viability. *C* control, *OX* oxLDL treatment; *TX* Triton-X100 detergent. Values presented are mean ± SD, n = 3–7.

**Figure 4:**
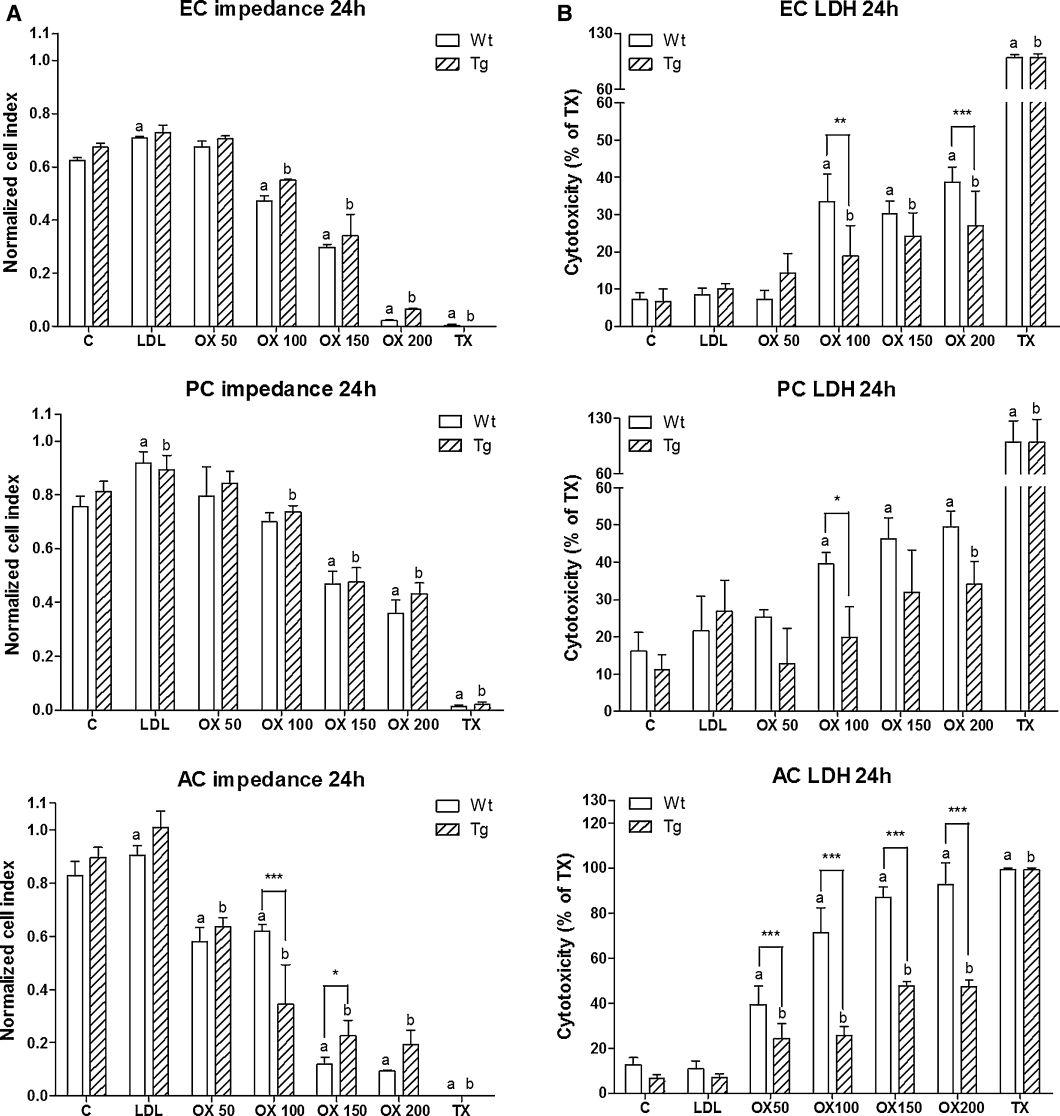
Effects of low density lipoprotein (LDL) and oxidized LDL on cell viability: impedance and lactate dehydrogenase (LDH) release. Effects of LDL or oxLDL treatment on the viability of cultured cells from wild type (Wt) and ApoB-100 transgenic (Tg) mice. **A** Normalized cell index reflect to the viability of the cells 24 h after treatment. Viability of brain endothelial cells (EC), pericytes (PC) and astroglia cells (AC) is presented after LDL or oxLDL treatment. **B** Lactate dehydrogenase release of the cells 24 h posttreatment. *C* control, *OX* oxLDL treatment, *TX* Triton-X100 detergent. Values presented are mean ± SD, n = 3–7. Statistical analysis: ANOVA followed by Dunnett and Bonferroni tests. Statistically significant differences: (*a*) compared to wild type control; (*b*) compared to transgenic control; *p < 0.05; **p < 0.01; ***p < 0.001 wild type compared to transgenic group.

Treatment with oxLDL resulted in cell damage in all three cell types. Impedance measurement showed that oxLDL exerted a dose- and time-dependent reduction in cell viability (Figures 3, 4). Elevated LDH release indicates cell membrane injury at 24 h in all treated groups (Figure 4). Astroglia cells proved to be the most sensitive to oxLDL treatments while pericytes were the least affected. Brain endothelial and glial cells from ApoB-100 transgenic mice showed smaller reduction in viability and membrane damage compared to their wild type controls. In contrast to oxLDL, LDL treatment elevated the impedance of cells reflecting a positive effect on viability and cell growth. No effect of LDL was found on LDH release confirming its non-toxic property. Primary pulmonary endothelial cells isolated from wild type and ApoB-100 transgenic mice were also damaged by oxLDL treatment measured by impedance, but no difference was seen between the sensitivity of the cells from the two groups of animals (Additional File 1: Figure S1).

### Effects of oxidized LDL on ROS and NO production in cultured brain endothelial cells, pericytes and glial cells from wild type and ApoB-100 transgenic mice

**Figure 5:**
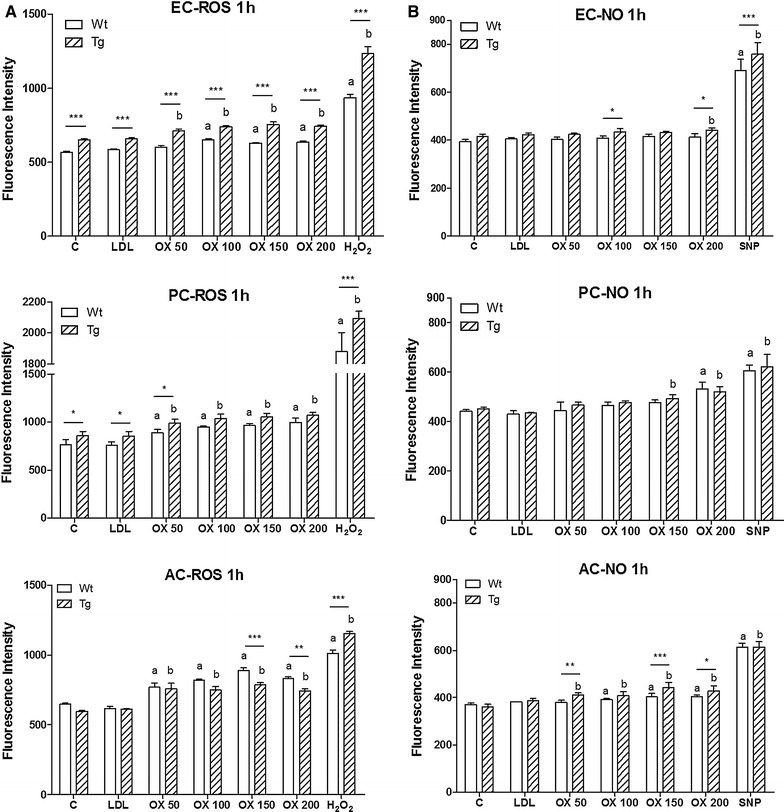
Effects of low density lipoprotein (LDL) and oxLDL treatment on ROS and NO production. Effects of LDL or oxLDL treatment on the reactive oxygen species (ROS) and nitric oxide (NO) production in cells from wild type (Wt) and ApoB-100 transgenic (Tg) mice. **A** ROS and **B** NO production of brain endothelial cells (EC), pericytes (PC) and astroglia cells (AC) is shown 1 h after treatment. Hydrogen peroxide (H_2_O_2_) served as positive control in the ROS assay, sodium nitroprusside (SNP) served as positive control in the NO assay. *C* untreated control, *OX* oxLDL treatment. Values presented are mean ± SD, n = 3–8. Statistical analysis: ANOVA followed by Dunnett and Bonferroni tests. Statistically significant differences: (*a*) compared to wild type control; (*b*) compared to transgenic control; *p < 0.05; **p < 0.01; ***p < 0.001 wild type compared to transgenic.

Treatment with oxLDL significantly increased ROS production in brain endothelial cells, pericytes and glial cells (Figure 5). In endothelial cells and pericytes, basal ROS production and ROS production after oxLDL treatment was higher in cells from ApoB-100 transgenic mice compared to wild type, but no such effect was seen for NO. NO production was only elevated at the highest treatment concentrations of oxLDL in endothelial cells and pericytes, but it resulted in an increase in NO production even from the lowest treatment concentration in astroglial cells (Figure 5). LDL treatment had no effect on ROS or NO production in these cultured cells.

### Effects of oxidized LDL on the barrier integrity of co-culture BBB models from wild type and ApoB-100 transgenic mice

**Figure 6:**
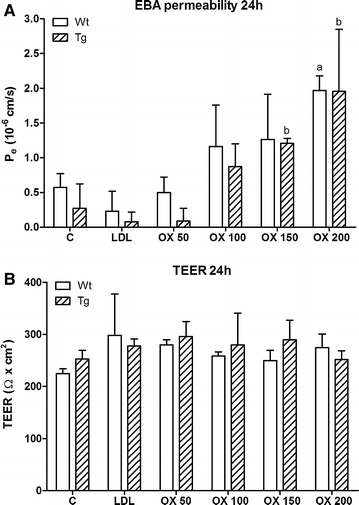
Effects of low density lipoprotein (LDL) and oxLDL on the barrier integrity of co-culture BBB models. Effects of LDL or oxLDL treatment on the barrier integrity of BBB models from wild type (Wt) and ApoB-100 transgenic (Tg) mice. Brain endothelial cells co-cultured with pericytes and astroglia cells were treated for 24 h with LDL or oxLDL. **A** Permeability of mouse brain endothelial cell layers for Evans blue labeled albumin (EBA) is expressed as endothelial permeability coefficient (Pe, cm/s). **B** Transendothelial electrical resistance (TEER) is expressed as Ω cm^2^. *C* untreated control, *OX* oxLDL treatment. Values presented are mean ± SD, n = 3. Statistical analysis: ANOVA followed by Dunnett and Bonferroni post-tests. Statistically significant differences: (*a*) *p* < 0.05 compared to wild type control; (*b*) *p* < 0.05 compared to transgenic control.

When cells reached 264 ± 33 Ω cm^2^ TEER value on the 4th day of co-culture, the effect of oxLDL treatment (24 h) was tested on the triple co-culture models of the BBB using Evans blue labeled albumin as marker molecule of transendothelial permeability (Figure 6). Low concentrations of oxLDL did not change the flux of albumin, but higher treatment concentrations (150 and 200 µg/ml) significantly elevated the albumin penetration across cell layers in both groups. No significant change was observed in the electrical resistance of the models between the control and the treatment groups (Figure 6). There was no difference between the resistance of the BBB models from wild type (262 ± 30 Ω cm^2^) or ApoB-100 transgenic (266 ± 37 Ω cm2) mice or in their albumin permeability (Figure 6).

**Figure 7:**
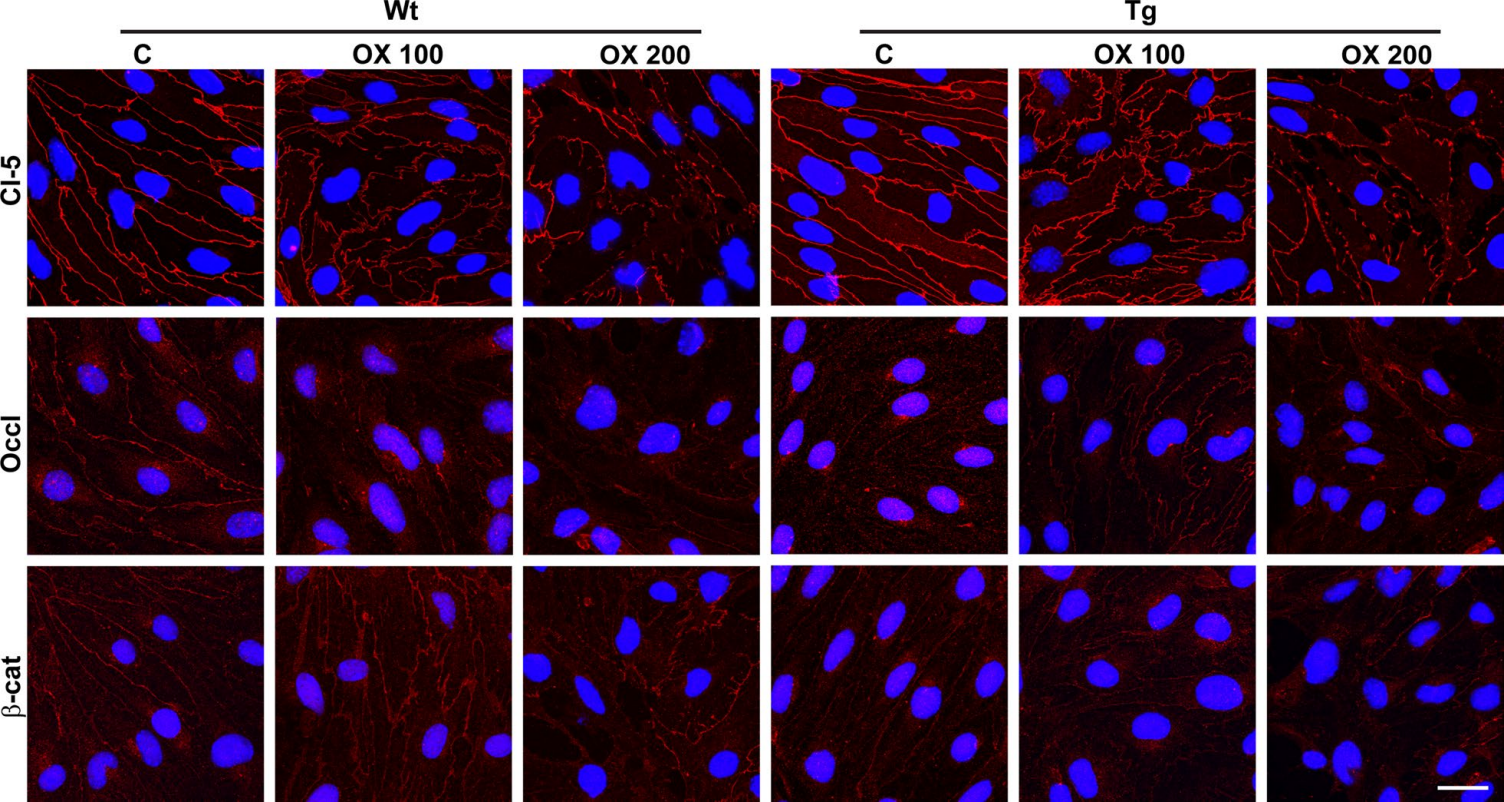
Effect of oxLDL on junctional stainings in cultured brain microvascular cells from wild type (Wt) and transgenic (Tg) mice. Immunostaining of brain endothelial cells for transmembrane junctional protein claudin-5 (Cl-5) and occludin (Occl), and cytoplasmic linker protein β-catenin (β-cat) after 100 and 200 µg/ml oxLDL treatment or in untreated controls (C). *Red color* junctional immunostaining. *Blue* cell nuclei. *Scale bar* 25 µm.

We treated primary brain endothelial cells from both groups of mice with two concentrations of oxLDL (100 and 200 µg/ml) and stained them for junctional proteins claudin-5, occludin and β-catenin (Figure 7). The junctional staining changed in oxLDL treated cells, both the intensity and localization was altered compared to control conditions indicating barrier disturbance. We found no labeling of cell nuclei with ethidium-homodimer-1 indicating no cell death, while all cell nuclei were labeled with bis-benzimide (H33343).

### Effects of oxidized LDL on membrane fluidity of brain endothelial cells from wild type and ApoB-100 transgenic mice

**Figure 8:**
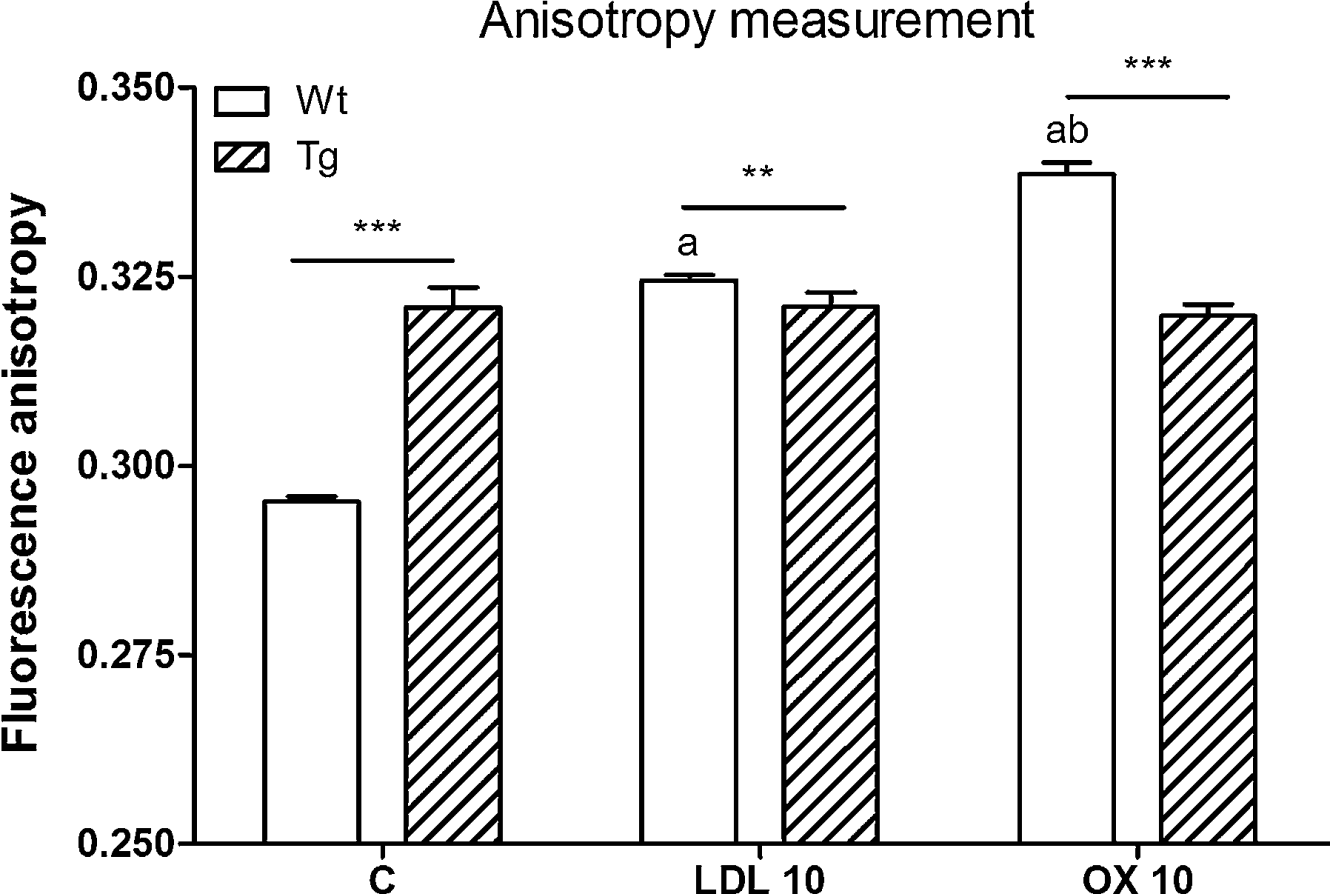
Effects of low density lipoprotein (LDL) and oxLDL on membrane fluidity of brain endothelial cells measured as anisotropy. Effects of oxLDL or LDL treatment on the membrane fluidity of cultured brain endothelial cells from wild type (Wt) and ApoB-100 transgenic (Tg) mice. Effects were measured by TMA-DPH fluorescence anisotropy on cell suspensions after overnight treatment with 10 µg/ml LDL or oxLDL treatment. *C* untreated control, *OX* oxLDL treatment. Values presented are mean ± SD, n = 3. Statistical analysis: ANOVA followed by Dunnett and Bonferroni post-tests. Statistically significant differences: (*a*) *p* < 0.001 compared to wild type control; (*b*) *p* < 0.001 compared to LDL treated wild type cells; *p < 0.05; **p < 0.01; ***p < 0.001 wild type compared to transgenic group.

Membrane fluidity of living brain endothelial cells was determined by the measurement of fluorescence anisotropy of the cationic membrane probe TMA-DPH (Figure 8). Anisotropy of both LDL and oxLDL treated wild type cells was elevated compared to the control. Treatment with oxLDL increased anisotropy more than the treatment with LDL, indicating higher membrane rigidity. The anisotropy of brain endothelial cells from ApoB-100 transgenic mice was significantly higher than that of wild type cells which was not changed by treatment with LDL or oxLDL. The membrane fluidizer benzyl alcohol quickly and greatly reduced the anisotropy (cells: 0.320 ± 0.011 vs. benzyl alcohol: 0.307 ± 0.011).

## Discussion

ApoB-100 transgenic mice fed cholesterol high diet have been used for a long while to study cardiovascular diseases [6]. Recently, cognitive impairment caused by dyslipidemia and/or hyperlipidemia was reported in this model [10, 28]. The BBB is the primary target of deteriorative changes caused by dyslipidemia and/or hyperlipidemia eventually leading to the development of neurodegeneration and dementia [29]. Brain capillary injury including morphological changes in brain endothelial and swelling of astrocytic endfeet were seen in hyperlipidemic transgenic mice after ischemic challenge [11]. The role of pericytes was also recently revealed in cerebrovascular pathology in hyperlipidemic mice [30]. Although, there is accumulating evidence of harmful effects of hyperlipidemia on the BBB, very limited information is available on the phenotypic, morphological and functional changes of the BBB at the cellular level. Therefore in the present study morphological phenotypes of cultured brain endothelial cells, pericytes and glial cells isolated from ApoB-100 transgenic and wild type mice were investigated.

ApoB-100 is expressed mainly in the liver, intestine and heart [31, 32]. However, the presence of ApoB-100 protein was demonstrated in the membrane microdomains of primary brain endothelial cells using mass spectrometry [33]. Here, we confirm this result and using immunohistochemistry we show that ApoB-100 is apparently present in brain endothelial cells. Although further experiments may be necessary to confirm this result, we suggest that ApoB-100 is synthesized in these cells. The transgenic construct contained the whole genomic ApoB-100 sequences (43 kb) including all the 29 introns and a 19 kb long 5′ and 14 kb long 3′ sequences providing all putative regulatory elements for correct tissue-specific gene expression [34]. Therefore, the expression of human ApoB-100 protein in transgenic mice reflects the endogenous expression of the mouse gene. Indeed, neither others nor we have experienced any ectopic expression using this transgenic construct [34]. Further supporting the present observation the cerebrovascular/perivascular localization of ApoB-100 in transgenic mice was demonstrated in our recent paper [10]. In addition to de novo synthesis, ApoB-100 uptake into brain endothelial cells can also be considered. In a parallel in vivo experiment we detected a nearly three-fold increase in the expression level of Lox1 gene in isolated microvessels from transgenic mouse brains using qPCR, which may indicate enhanced transport of lipoproteins into the cells of the BBB (Lénárt et al. 2015 unpublished). Several groups have used the ApoB or ApoB-binding domain for brain targeting of nanoparticles across the BBB [35, 36], which also indicates an active uptake/transport of ApoB-100 in brain endothelium in vivo. A long-term saturated fat diet in mice induces neurovascular inflammation and brain capillary dysfunction measured by the brain extravasation of plasma proteins ApoB and immunoglobulin G [37].

Using immunofluorescence, no obvious morphological differences were detected between genotypes concerning glial cells, brain pericytes and endothelial cells. While no change was observed for transmembrane junctional proteins, the fluorescence intensity of the junctional cytoplasmic linker proteins, ZO-1 and β-catenin was higher in brain endothelial cells derived from transgenic mice but resistance values were not different between BBB models from the two groups of mice. Based on our data, the intensity differences in ApoB-100, ZO-1 and β-catenin immunohistochemistry in cultured brain endothelial cells from ApoB-100 transgenic mice as compared to wild type may not have functional significance for barrier integrity of the BBB model.

Hyperlipidemia causes oxidative stress which damages cerebral capillaries [10]. Therefore, the sensitivity of primary cells to oxLDL treatment was assessed. OxLDL accumulates in the atherosclerotic lesions of humans [38] and is also present in ApoE^−/−^ mice [39]. OxLDL was identified as initiator of endothelial dysfunction [40]. We found that the sensitivity of brain endothelial cells, pericytes and glial cells to oxLDL (50–200 µg/ml) treatment is largely different. Astrocytes and endothelial cells were found to be the more vulnerable to oxLDL treatment compared to pericytes. A dose-dependent reduction in cell impedance was observed for all treated cell types. Our observation is consistent with the results of previous reports describing the toxic effect of oxLDL on brain endothelial cells [41] and retinal pericytes [42]. Primary pulmonary endothelial cells, which do not form a tight barrier, were also damaged by oxLDL treatment. Furthermore, oxLDL caused barrier dysfunction and alterations in junctional morphology of brain endothelial cells in triple co-culture BBB models in accordance to literature data on mouse brain monolayers [43, 44]. Our results indicate that the transcellular transport of albumin is increased after oxLDL treatment. Similar effects, no change in paracellular transport but elevation in albumin transfer, were seen after hypoxia [45] or histamine treatment [46] in BBB co-culture models. There was no difference between the basal barrier function of the BBB models from wild type and ApoB-100 transgenic mice, in concordance with the similarity of morphology and immunostaining intensity of transmembrane tight junctional proteins in brain endothelial cells from both groups.

Interestingly, transgenic brain endothelial cells and astrocytes showed smaller reduction in viability and increased resistance to membrane damage compared to wild-type cells indicating certain protection against oxLDL-induced cytotoxicity in cultured cells derived from ApoB-100 transgenic animals. No difference was seen between the sensitivity of peripheral (pulmonary) endothelial cells from the two groups of mice. To reveal the possible mechanism behind this phenomenon, NO and ROS production was monitored in these cells during oxLDL treatment. OxLDL induces production of reactive oxygen and nitrogen species and the resulting oxidative stress leads to endothelial dysfunction and consequently to atherosclerosis and cardiovascular diseases [47]. A significant increase in ROS production was detected in all cell types after oxLDL treatment. These results are in agreement with previous work of Chang et al. [48] who also showed a similar increase in ROS production after oxLDL treatment in mouse brain endothelial cells. ROS production induced by oxLDL in human umbilical vein endothelial cells was increased via the enhanced activity of NADPH oxidase and reduced activity of superoxide dismutase and catalase [49]. Mitochondrial damage due to increased reactive oxygen and nitrogen species formation in response to oxLDL treatment was also reported in bovine aortic endothelial cells [50]. Interestingly, in endothelial cells and pericytes, basal ROS production and ROS production after oxLDL treatment were higher in cells from ApoB-100 transgenic mice compared to wild type indicating that the protective effect seen in viability studies is not mediated by lower production of ROS. OxLDL promotes the generation of superoxide that reduces the activity of endothelial nitric oxide synthase thus decreasing the amount of NO in peripheral endothelial cells [51]. In the present study, high concentrations of oxLDL increased the production of NO in all three cell types of the brain microvasculature. These are the first data on cultured brain endothelial cells, pericytes and glial cells and the results are different from observations on the effect of oxLDL on peripheral endothelial cells. Although no comparative data are available, cells related to cerebral vasculature may differ from peripheral endothelial cells in several aspects including NO production. NO plays a central role in cerebrovascular regulation and all cells of the neurovascular unit contribute to its production by a complex network of regulation [52].

Lipid enriched membrane microdomains in brain endothelial cells contribute to BBB functions including cell polarity, paracellular barrier formation, transport pathways and signalling [33]. These microdomains are enriched in cholesterol and sphingolipids [53]. Membrane cholesterol and rafts are modified by oxLDL [54]. Oxysterols, responsible for the toxic effect of highly oxidized LDL, are incorporated into the plasma membrane thus perturbing the distribution of cholesterol-rich membrane microdomains [55]. Changes in plasma membrane fluidity may reflect disturbances in membrane cholesterol and rafts, therefore living brain endothelial cells were examined for TMA-DPH fluorescence anisotropy for the first time. Treating wild type brain endothelial cells with native or oxLDL resulted in remarkable increase in anisotropy, indicating higher level of membrane rigidity. The membrane of brain endothelial cells from transgenic animals was significantly stiffer than in wild type mice. This apparent high membrane rigidity of transgenic cells was not changed further by treatment. In agreement with our results, oxLDL also reduced the fluidity of the outer membrane layer of platelets [56]. While it is known that changes in plasma membrane fluidity and lipid rafts are linked to cell death [57], further experiments are needed to clarify the importance of the increased membrane rigidity in cells from ApoB-100 transgenic animals.

## Conclusion

The morphological and functional properties of cultured brain endothelial cells, pericytes and glial cells from ApoB-100 transgenic mice were characterized and compared to wild type cells for the first time. The presence of ApoB-100 was confirmed in brain endothelial cells, while no gross morphological change was observed between wild type and transgenic cells. Oxidized but not native LDL exerted dose-dependent toxicity in all three cell types, induced barrier dysfunction and increased ROS production in both genotypes. A partial protection from oxLDL toxicity was seen in brain endothelial and glial cells from ApoB-100 transgenic mice. Increased membrane rigidity was measured in brain endothelial cells from ApoB-100 transgenic mice and in LDL or oxLDL treated wild type cells indicating alterations in lipid composition. To understand the link between membrane fluidity and increased growth kinetics and partial protection in ApoB-100 transgenic cells related brain microvasculature further experiments are needed.

## Additional files

Additional file 1:
**Description of data.** Isolation method, characterization, immunohistochemistry and cell viability after oxLDL treatment of primary mouse pulmonary endothelial cells from wild type and ApoB-100 transgenic mice.

## Abbreviations

ApoB-100: apolipoprotein B-100
BBB: blood–brain barrier
BSA: bovine serum albumin
DAF-FM: 4-amino-5-methylamino-2′,7′-difluorofluorescein diacetate
DCFDA: chloromethyl-dichloro-dihydro-fluorescein diacetate
DMEM: Dulbecco’s modified Eagle medium
FBS: fetal bovine serum
GFAP: glial fibrillary acidic protein
H_2_O_2_: hydrogen peroxide
LDH: lactate dehydrogenase
LDL: low density lipoprotein
oxLDL: oxidized low density lipoprotein
NADPH: nicotinamide adenine dinucleotide phosphate
NO: nitric oxide
PBS: phosphate buffered saline
ROS: reactive oxygen species
SD: standard deviation
α-SM actin: α-smooth muscle actin
SNP: sodium nitroprusside
TEER: transendothelial electrical resistance
TMA-DPH: 1-(4 trimethylammoniumphenyl)-6-phenyl-1,3,5-hexatriene
VLDL: very low density lipoprotein
ZO-1: zonula occludens protein-1
