# Supplementary material for: Cultured cells of the blood–brain barrier from apolipoprotein B-100 transgenic mice: effects of oxidized low-density lipoprotein treatment
Source: Fluids Barriers CNS. 2015 Jul 17;12:17. doi: 10.1186/s12987-015-0013-y (PMC4504453; doi:10.1186/s12987-015-0013-y)
Supplement: Additional file 1: — Description of data. Isolation method, characterization, immunohistochemistry and cell viability after oxLDL treatment of primary mouse pulmonary endothelial cells from wild type and ApoB-100 transgenic mice. [file 12987_2015_13_MOESM1_ESM.pdf]

Additional file 1.

Supplementary material to:

Cultured cells of the blood-brain barrier from apolipoprotein B-100 transgenic mice: effects of oxidized LDL treatment

Nikolett Lénárt[1#]*, Fruzsina R. Walter[2]*, Alexandra Bocsik[2], Petra Sántha[2], Melinda E. Tóth[1], András Harazin[2], Andrea E. Tóth[2], Zsolt Török[3], Ana-Maria Pilbat[3], László Vígh[3], László G. Puskás[4], Miklós Sántha[1]*, Mária A. Deli[2]*

[1]Laboratory of Animal Genetics and Molecular Neurobiology, Institute of Biochemistry, Biological Research Centre, Hungarian Academy of Sciences, H-6720, Temesvári krt. 62, Szeged, Hungary

[2]Biological Barriers Research Group, Institute of Biophysics, Biological Research Centre, Hungarian Academy of Sciences, Szeged, Hungary

[3]Laboratory of Molecular Stress Biology, Institute of Biochemistry, Biological Research Centre, Hungarian Academy of Sciences, Szeged, Hungary

[4]Laboratory of Functional Genomics, Laboratories of Core Facilities, Biological Research Centre, Hungarian Academy of Sciences, Szeged, Hungary

#current affiliation: Laboratory of Molecular Neuroendocrinology, Institute of Experimental Medicine, Hungarian Academy of Sciences, Budapest, Hungary

* authors contributed equally to this manuscript

Correspondence to:
Maria A. Deli (Telephone: +36 62 599602; Fax: +36 62 433133;
E-mail: deli.maria@brc.mta.hu)

Methods

Materials

All reagents were purchased from Sigma-Aldrich Ltd., Hungary, unless otherwise indicated.

Isolation of mouse pulmonary endothelial cells

Primary mouse pulmonary endothelial cells (MPECs) were isolated from 2-week-old wild type and ApoB-100 transgenic mice based on the method of Sobczak et al. [1]. Lungs were excised under sterile conditions and collected in ice-cold Dulbecco's modified Eagle's medium (DMEM). Lung lobes were cut to small pieces by sterile scissors. Tissue samples were digested by collagenase-dispase (1 mg/ml; Roche, Switzerland) in DMEM containing DNase I (1 mg/ml; Roche, Switzerland) at 37°C for 45 min on a horizontal shaker. Then tissue suspensions were homogenized by a 20 ml syringe connected to a 14 g cannula and passed through a cell strainer (40 µm pore size, Millipore, USA) and washed with DMEM. After centrifugation (400 g, 5 min) pellets were resuspended in 3 ml phosphate buffer containing 0.1 % bovine serum albumin (BSA-PBS). Anti-CD31 (BioLegend, USA) antibody-conjugated Dynabeads (LifeTechnologies, USA) were prepared as recommended in the protocol and added to the cell suspensions. Tubes were kept tumbling at room temperature for 12 minutes. Then cell suspensions were transferred to microcentrifuge tubes and the beads were sedimented using Magnetic Particle Concentrator (LifeTechnologies, USA), supernatants were removed, then beads were washed by 0.1 % BSA-PBS for 5 times. Immunoselected cells were plated on 2 % gelatin coated Petri dishes (35 mm; Falcon; BD Biosciences, USA). MPECs were kept in MCDB131 medium (PAN-Biotech, Germany) containing 15 % fetal bovine serum, hydrocortisone (1.4 µM), HEPES (10 mM), acid ascorbic (5 µg/mL), chemically defined lipid concentrate (100x, Life Technologies, USA), insulin (5 µg/ml), transferrin (5 µg/ml), sodium selenite (5 ng/ml), heparin (100 µg/ml), glutamax (100 x, Life Technologies, USA), basic fibroblast growth factor (2 ng/ml; Roche, Switzerland), epidermal growth factor (2 ng/ml), gentamycin (50 µg/mL) and penicillin-streptomycin (50 µg/ml). Medium change was performed every second day, and on the sixth day of culture cells were passaged for real time impedance measurements and immunohistochemistry. MPEC isolation was repeated twice with same results.

Cell viability of primary mouse pulmonary endothelial cells after oxLDL treatment

Viability of MPECs after oxLDL treatment (200 μg/ml) was tested using the impedance-based cell electronic sensing (ACEA Biosciences, Inc., USA).

Immunohistochemistry of mouse primary pulmonary endothelial cells

Morphological characterization of MPECs was performed using immunohistochemical staining. The staining protocol for claudin-5, occludin, zonula occludens protein 1 (ZO-1) and β-catenin was identical to the one used for primary brain endothelial cells. Pulmonary endothelial cells were also labeled for VE-cadherin using goat-anti-VE-cadherin (1:70, Santa Cruz, USA) as primary and anti-goat Alexa Fluor 488 secondary (1:500, Jackson ImmunoResearch Labs, USA) antibody.

Results

Characterization and cell viability of mouse primary pulmonary endothelial cells

We successfully isolated and cultured MPECs from both wild type and ApoB-100 transgenic mice. Cells from both groups were immunolabeled for several junctional proteins. In contrast to primary brain endothelial cells MPECs did not stain for tight junctional proteins claudin-5 and occludin indicating weak barrier properties. A positive staining was observed for adherens junctional protein VE-cadherin similarly to the work of Sobczak et al. [1] and also for junctional associated proteins β-catenin and ZO-1 (Figure S1A). Confluent cultures of MPEC treated with oxLDL (200 μg/ml) showed reduced impedance indicating reduced cell viability. The effect of oxLDL seen on pulmonary endothelial cells was similar to rat BBB cells. However there was no difference in the sensitivity of pulmonary endothelial cells to oxLDL treatment from wild type and ApoB-100 transgenic mice (Figure S1B,C,D).

Figure S1

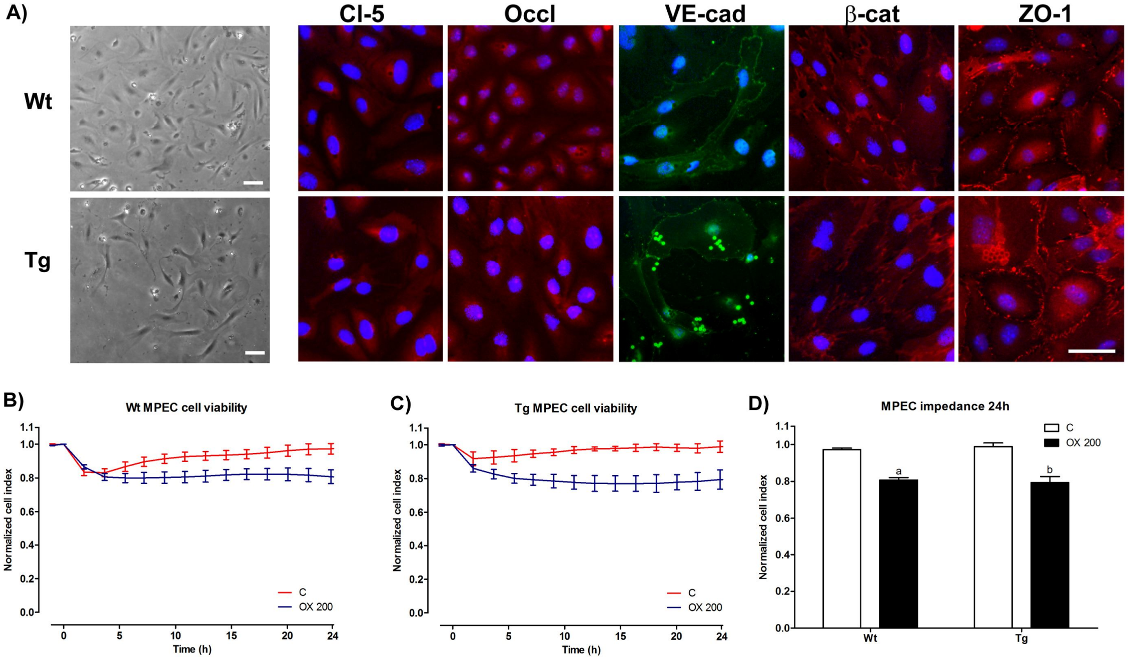

Figure S1 Characterization of cultured mouse pulmonary endothelial cells (MPECs) and viability after oxLDL treatment. Pulmonary endothelial cells were isolated from wild type (Wt) and ApoB-100 transgenic (Tg) mice. **A:** Phase contrast microscopy pictures of primary MPEC cultures. Scale bar: 100 µm. Immunostaining of MPECs for transmembrane junctional protein claudin-5 (Cl-5) and occludin (Occl), adherens junctional protein VE-cadherin and cytoplasmic linker proteins β-catenin (β-cat) and zonula occludens-1 (ZO-1). Blue: cell nucleus. Green dots (Tg MPEC VE-cadherin staining): Dynabeads. Red dots (Tg MPEC ZO-1 staining): Dynabeads. Scale bar: 50 µm. **B, C, D:** Effects of oxLDL treatment (200 µg/ml) on MPEC cell viability. C: control; OX: oxLDL treatment. Values presented are means ± SD, n = 8-16. Statistical analysis: ANOVA followed by Bonferroni tests. Statistically significant differences: a, compared to wild type control; b, compared to transgenic control ($p<0.001$).

Reference

1. Sobczak M, Dargatz J, Chrzanowska-Wodnicka M: **Isolation and culture of pulmonary endothelial cells from neonatal mice.** *J Vis Exp* 2010,**46:**pii: 2316.
